# HiGLDP: a hierarchical graph neural network for predicting lncRNA-disease associations through multi-omic integration

**DOI:** 10.1186/s12915-026-02557-z

**Published:** 2026-02-20

**Authors:** Yongtian Wang, Zhiyuan Wang, Tao Wang, Jialu Hu, Zhuhong You, Jiajie Peng

**Affiliations:** School of Computer Science, Northwestern Polytechnical University, Xi’an, 710072 China

**Keywords:** LncRNA, Disease, Multi-omic integration, Graph neural network, Stratified interaction graph

## Abstract

**Background:**

Long noncoding RNAs (lncRNAs) have emerged as crucial regulators in the pathogenesis of complex human diseases. Despite significant advances, identifying disease-associated lncRNAs remains challenging due to the vast noncoding transcriptome and the complexity of lncRNA interaction networks.

**Results:**

We propose HiGLDP, a computational framework for predicting lncRNA-disease associations through the integration of multi-omic data and advanced graph neural network techniques. HiGLDP constructs comprehensive similarity networks for lncRNAs and diseases using genomic, transcriptomic, and proteomic information, which are refined using random walk with restart (RWR) and denoising autoencoders (DAE). The bipartite lncRNA-disease association network is transformed into an interconnected graph with relationship nodes, while an association feature graph is constructed based on cosine similarity. A hybrid graph neural network architecture combining graph convolutional networks (GCN) and graph attention networks (GAT) is employed to capture both local and global graph structures, followed by a multilayer perceptron (MLP) for association classification. Comprehensive evaluations demonstrate that HiGLDP achieves superior predictive performance, accuracy, and robustness compared with existing methods. Case studies further validate its effectiveness in identifying novel lncRNA-disease associations.

**Conclusions:**

HiGLDP provides a robust and interpretable computational framework for lncRNA-disease association prediction. By integrating multi-omic information with hybrid graph learning, it offers valuable insights into lncRNA-disease interactions and represents a meaningful advancement in predictive modeling in this field.

**Supplementary Information:**

The online version contains supplementary material available at 10.1186/s12915-026-02557-z.

## Background

In recent years, the burgeoning field of long noncoding RNAs (lncRNAs) has unveiled a new horizon in understanding complex human diseases [1, 2]. LncRNAs, transcending the traditional view of noncoding sequences as mere genomic placeholders, have emerged as pivotal players in disease pathology through their intricate regulation of gene expression [3, 4]. The realization that lncRNAs contribute to the nuanced regulation of cellular processes accentuates their significance in disease etiology, particularly in the realm of complex human diseases [5, 6]. This burgeoning interest is underpinned by evidence demonstrating how mutations and dysregulation of lncRNAs are intimately linked to the progression and manifestation of various diseases, thereby positioning lncRNAs as both potential diagnostic markers and therapeutic targets [7].

However, the path to harnessing the full potential of lncRNAs in disease management is fraught with challenges [8]. A primary obstacle lies in the identification of disease-associated lncRNAs amid the vast expanse of the noncoding transcriptome. Despite the acceleration of lncRNA discovery through high-throughput sequencing technologies, the functional characterization of these entities remains in its infancy [9, 10]. The complexity is further compounded by the multifaceted nature of lncRNA interactions, which span across diverse biological pathways and molecular mechanisms, making the prediction and validation of lncRNA-disease associations a challenging problem [11]. Moreover, the heterogeneity of diseases themselves, with their varying genetic and environmental underpinnings, adds another layer of complexity to discerning the specific roles of lncRNAs in disease pathogenesis [12].

Addressing these challenges necessitates innovative computational models that can adeptly navigate the intricacies of ncRNA functionality and disease association [13–16]. Such models must not only integrate multiple biological datasets, encompassing genomic, transcriptomic, and proteomic dimensions but also employ sophisticated algorithms capable of discerning the subtle patterns that link specific lncRNAs to disease processes [17]. The SIMCLDA method [18], utilizing inductive matrix completion and integrating multiple biological datasets, stands out for its capacity to predict potential associations with newly identified lncRNAs, illustrating the method’s adeptness in dealing with sparse data. Meanwhile, GCRFLDA [19] integrates GCNs with conditional random fields (CRFs), a sophisticated blend that excels in capturing both the broad structures and finer contextual similarities within biological interactions. DMFLDA [20] applies deep learning to matrix factorization, revealing the nuanced, nonlinear relationships between lncRNAs and diseases. gGATLDA is a computational method to predict LDAs based on graph-level graph attention network [21]. It constructs the subgraphs of lncRNA and disease and uses a graph neural network (GNN) model to predict lncRNA-disease potential association scores. GMCLDA [22] is a method based on geometric matrix completion. It introduces association patterns among functionally similar lncRNAs and phenotypically similar diseases and then estimates the missing entries of the association matrix based on geometric matrix completion model. VGAELDA is a representation learning model based on variational inference and graph autoencoder [23]. VGAELDA integrates two types of graph autoencoders. Variational graph autoencoders (VGAE) infer representations from the features of lncRNAs and diseases, while graph autoencoders propagate labels through known lncRNA-disease associations. Alternate training with the variational expectation maximization algorithm was performed to enhance VGAELDA’s ability to extract efficient low-dimensional features from high-dimensional features. Lastly, GANLDA [24] introduces a novel use of graph attention networks to dynamically weigh the relevance of different nodes, effectively reducing noise through PCA and illustrating superior predictive performance. Collectively, these computational models have significantly advanced our understanding of the complex interplay between lncRNAs and diseases. They each bring forth new methodologies aimed at enhancing prediction accuracy.

Despite the significant advancements in computational models for predicting lncRNA-disease associations, several critical challenges persist. Methods such as SIMCLDA, which utilize inductive matrix completion, are heavily reliant on high-quality, extensive datasets. This dependency poses significant challenges in data-limited scenarios, leading to suboptimal predictive performance. Similarly, the GCRFLDA model, which combines GCNs with conditional random fields (CRFs), faces practical challenges due to its computational intensity and complexity. The sophisticated algorithms require substantial computational resources, limiting their feasibility for large-scale studies or real-time applications. Deep learning-based approaches like DMFLDA, while powerful in uncovering nonlinear relationships, often suffer from a lack of interpretability. The “black-box” nature of these models makes it difficult to understand the derivation of specific predictions, which is critical for clinical applications. Additionally, the performance of these models is heavily influenced by the availability of rich interaction data. GANLDA models introduce innovative mechanisms for feature construction and relevance weighting, but their complexity can lead to overfitting and challenges in interpreting results.

In this study, we propose HiGLDP, a hierarchical graph learning framework for predicting lncRNA–disease associations. HiGLDP addresses the limitations of existing methods through three key innovations. First, it systematically integrates genomic, transcriptomic, and proteomic data to construct comprehensive similarity networks for both lncRNAs and diseases, from which robust multi-omic representations are learned using the random walk with restart (RWR) and denoising autoencoder (DAE) modules. Second, HiGLDP reformulates the traditional bipartite graph into a stratified interaction graph, in which each lncRNA-disease pair is modeled as an association node, and two complementary graphs, the interconnected graph and the association feature graph, jointly capture topological and functional dependencies. Third, a hybrid GCN–GAT architecture with an attention-based fusion mechanism adaptively balances local and global information, improving predictive accuracy while enhancing interpretability. Together, these innovations establish HiGLDP as a robust, scalable, and biologically interpretable framework that advances lncRNA–disease association prediction beyond conventional graph learning approaches.

## Results

### Overview

**Fig. 1:**
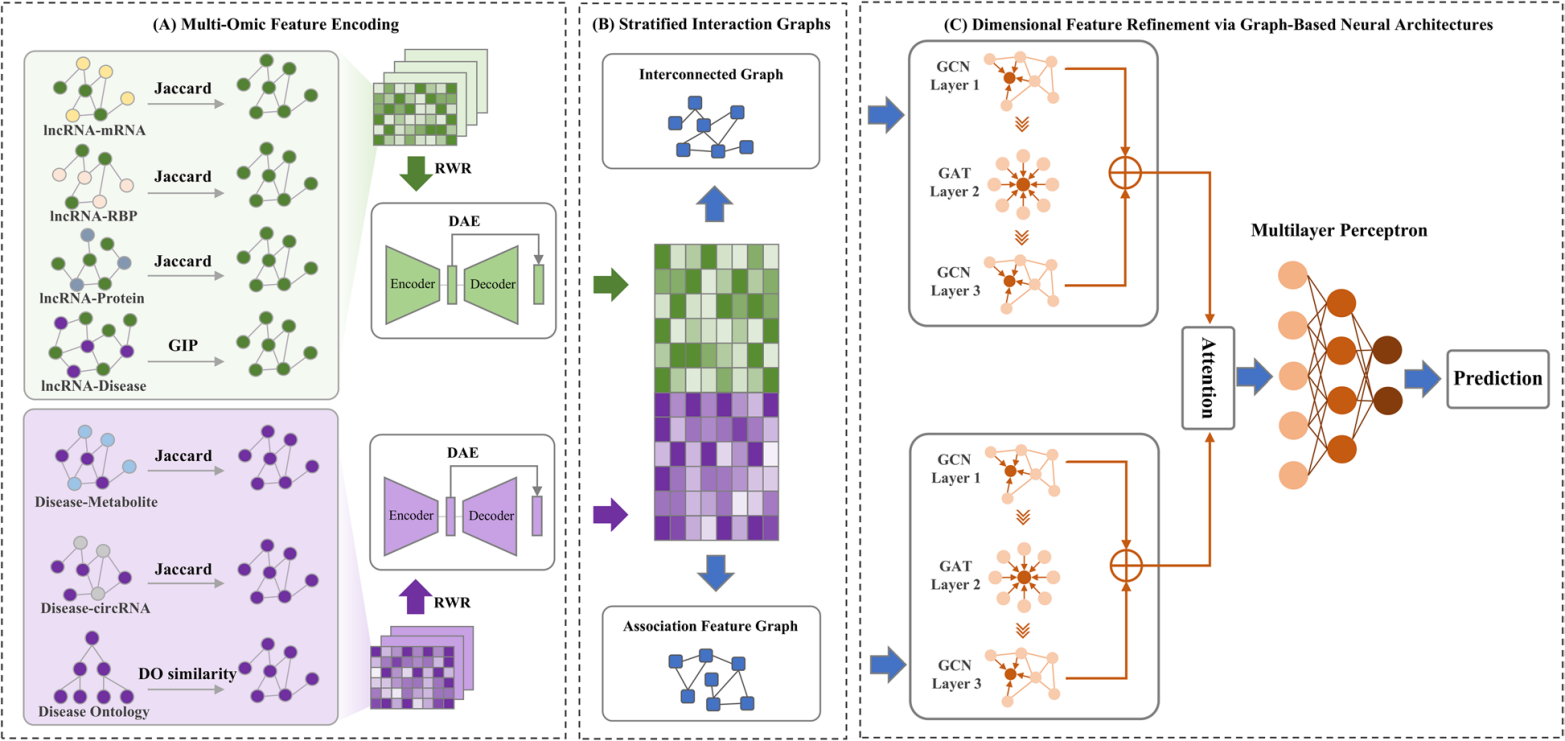
Overview of the HiGLDP model for predicting lncRNA-disease associations. **A** Construction of similarity networks for lncRNAs and diseases using Jaccard and GIP kernels for lncRNAs and FNSemSim for diseases. These similarity matrices are refined using RWR and DAE. **B** The bipartite graph of lncRNA-disease associations is transformed into an interconnected graph, while features from lncRNAs and diseases are concatenated to form an association feature graph using cosine similarity. **C** Extraction of key features from these graphs using a hybrid graph neural network with GCN and GAT, followed by classification with a MLP to predict lncRNA-disease associations

In this study, we develop HiGLDP, a computational model designed to predict lncRNA-disease associations through multi-omic integration and advanced graph neural network techniques (Fig. 1). We begin by constructing comprehensive similarity networks for lncRNAs and diseases using multiple biological datasets, including genomic, transcriptomic, and proteomic data. Specifically, we calculate lncRNA similarity using Jaccard and Gaussian interaction profile (GIP) kernels, while disease similarity is assessed using the FNSemSim method, which was developed before. These similarity networks are refined using RWR method, followed by feature extraction with DAE to generate robust representations. Based on representations of lncRNAs and diseases as well as their associations, we construct a stratified interaction graph. Specifically, the bipartite graph of lncRNA-disease associations is then transformed into an interconnected graph by defining relationship nodes that encapsulate lncRNA-disease associations. Meanwhile, features from lncRNAs and diseases are concatenated, and an association feature graph is constructed using cosine similarity. Considering both local and global graph structures, we employ a hybrid graph neural network architecture composed of GCN and GAT to extract key features from these graphs. The attention mechanism enhances interpretability by dynamically weighing the relevance of different features. Finally, MLP is used as the classification model, with the input being the representation vector of the relationship node and the output being the probability of association between lncRNA and disease. This integrative approach aims to improve the accuracy and interpretability of lncRNA-disease predictions, addressing key challenges in the field.

### Metrics

To assess the predictive performance of the HiGLDP model in identifying associations between lncRNA and disease pairs, we employed both fivefold and tenfold cross-validation techniques. To scientifically compare the performance of various models, we utilized six evaluation metrics: AUC, AUPR, precision, recall, F1 score, and Matthews correlation coefficient (MCC). MCC can be calculated as follows:

1 $$\mathrm{MCC}=\frac{\mathrm{TP}\times \mathrm{TN}-\mathrm{FP}\times \mathrm{FN}}{\sqrt{\left(\mathrm{TP}+\mathrm{FP}\right)\left(\mathrm{TP}+\mathrm{FN}\right)\left(\mathrm{TN}+\mathrm{FP}\right)\left(\mathrm{TN}+\mathrm{FN}\right)}}$$

where TP is the number of true positives, TN is the number of true negatives, FP is the number of false positives, and FN is the number of false negatives. The horizontal axis of the curve represents the false-positive rate (FPR), also known as 1-specificity, while the vertical axis represents the true-positive rate (TPR), also known as sensitivity.

Based on different thresholds, we can use the receiver operating characteristic curve (ROC) and AUC to assess the lncRNA-disease association capability of our proposed model. To balance the samples of known and unknown lncRNA-disease associations, we also draw the precision recall (PR) curve and calculated the area under the PR curve (AUPR). AUPR is the area under precision-recall curve. The horizontal ordinate of the curve is the recall⁠, and the longitudinal coordinate is the precision⁠.

The value of AUPR is also obtained by plotting the precision-recall curve and calculating the total area under the curve. The AUPR is more informative than AUC for the imbalanced association samples. The F1 score is a measure of accuracy and is particularly useful for imbalanced datasets. It is the harmonic mean of precision and recall, providing a single metric that balances both the false positives and false negatives. A higher F1 score indicates better performance of the model, as it considers both the precision and recall.

### Evaluating the performance of this model

**Fig. 2:**
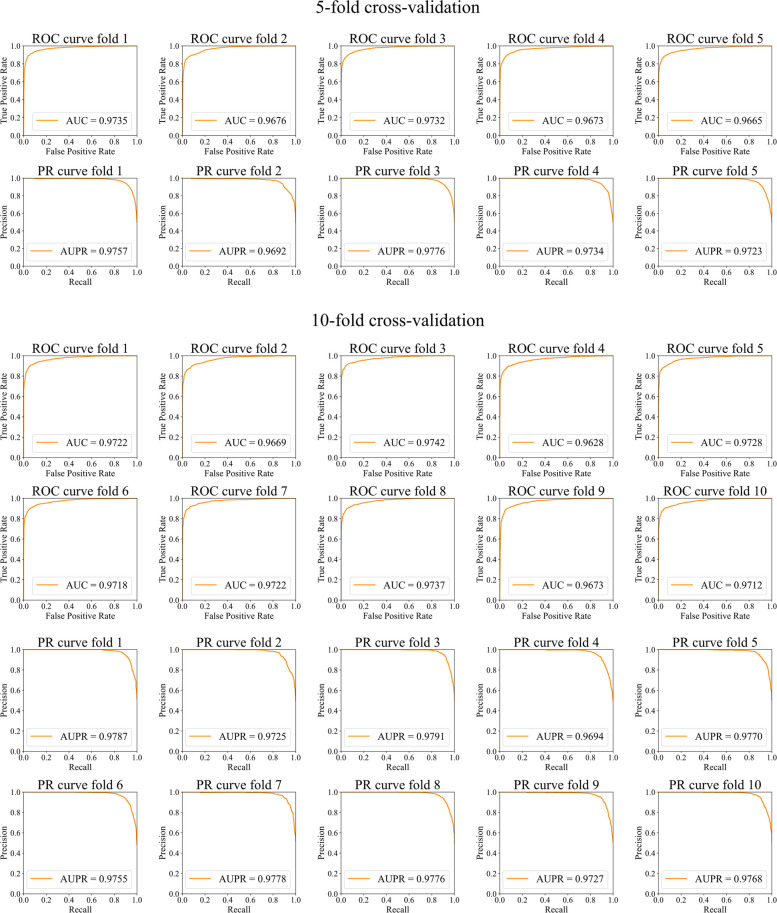
Comprehensive performance evaluation of HiGLDP

HiGLDP, assessed using both fivefold and tenfold cross-validation methods, exhibited robust predictive performance. The model achieved an average precision of 0.9366 and recall of 0.9005 in the fivefold cross-validation and an average precision of 0.9214 and recall of 0.9037 in the tenfold cross-validation (Table 1). These results correspond to average F1 scores of 0.9181 and 0.9115, respectively. The model’s reliability is further highlighted by strong Matthews correlation coefficients (MCC) of 0.8400 and 0.8262, demonstrating a significant correlation between the observed and predicted classifications. The model also achieved stable and impressive scores in area under the ROC curve (AUC) and precision-recall curve (AUPR) by using a fivefold cross-validation approach, with averages of 0.9696 and 0.9736, and a tenfold cross-validation approach, with averages of 0.9705 and 0.9757, respectively. This showcases its excellent ability to distinguish between positive and negative classes. The ROC curves depicted in Fig. 2 illustrate consistent performance across different data splits, characterized by high AUC values that demonstrate strong discrimination between true-positive and false-positive rates. Similarly, the PR curves underscore the model’s ability to sustain high precision across a range of recall levels, indicating its effectiveness in identifying true-positive cases across different classification thresholds. These findings underscore the efficacy and reliability of the HiGLDP model in predicting lncRNA-disease associations. The results under the fivefold and tenfold cross-validation showed highly consistent performance, with differences in AUC and AUPR of less than 0.01, indicating that the model remains stable across different training–testing splits. The slightly higher scores observed in the tenfold setting suggest that HiGLDP can effectively leverage additional training data without overfitting, reflecting strong generalization and convergence properties.

**Table 1:** The k-fold cross-validation testing results of HiGLDP

Round	AUC	AUPR	Precision	Recall	F1 score	MCC
**Fivefold cross-validation**						
**1**	0.9735	0.9757	0.9345	0.9095	0.9218	0.8465
**2**	0.9676	0.9692	0.9419	0.8898	0.9151	0.8355
**3**	0.9732	0.9776	0.9200	0.9192	0.9196	0.8382
**4**	0.9673	0.9734	0.9506	0.8822	0.9151	0.8386
**5**	0.9665	0.9723	0.9362	0.9020	0.9187	0.8414
**Average**	0.9696	0.9736	0.9366	0.9005	0.9181	0.8400
**tenfold cross-validation**						
**1**	0.9722	0.9787	0.9578	0.8849	0.9199	0.8446
**2**	0.9669	0.9725	0.9541	0.8756	0.9132	0.8373
**3**	0.9742	0.9791	0.8376	0.9568	0.8933	0.7817
**4**	0.9628	0.9694	0.9002	0.9052	0.9027	0.8090
**5**	0.9728	0.9770	0.9066	0.9215	0.9140	0.8274
**6**	0.9718	0.9755	0.9300	0.9028	0.9162	0.8409
**7**	0.9722	0.9778	0.9182	0.9101	0.9142	0.8235
**8**	0.9737	0.9776	0.9099	0.9116	0.9108	0.8236
**9**	0.9673	0.9727	0.9678	0.8628	0.9123	0.8371
**10**	0.9712	0.9768	0.9320	0.9054	0.9185	0.8368
Average	0.9705	0.9757	0.9214	0.9037	0.9115	0.8262

### Comparative efficacy with benchmark models

**Fig. 3:**
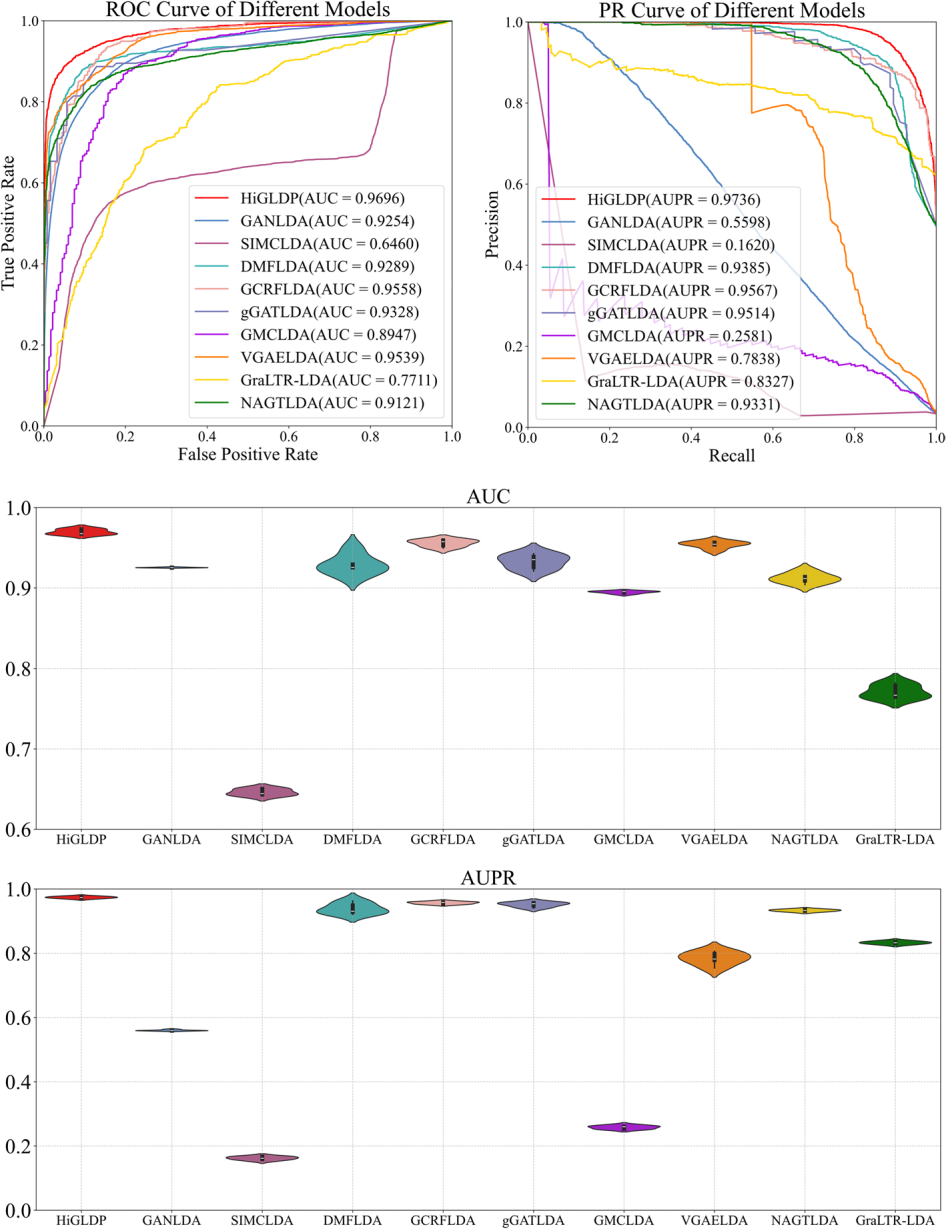
Comparative performance analysis of computational models in predicting LncRNA-disease associations. It offers a thorough comparison of multiple computational models using a fivefold cross-validation approach. The ROC curves depict the true-positive rates plotted against false-positive rates. The AUC violin and AUPR violin plots illustrate the distribution and peak performance of each model. Individual data values for all performance metrics are provided in Additional file 1

To demonstrate the superiority of HiGLDP, we compared it with seven baseline methods by fivefold cross-validation on benchmark datasets. These seven methods include GANLDA, SIMCLDA, DMFLDA, GCRFLDA, gGATLDA, GMCLDA, VGAELDA, GraLTR-LDA [25], and NAGTLDA [26]. When assessed using a fivefold cross-validation method, HiGLDP’s AUC was clearly superior. Table 2 showed the compared results; the AUC (0.9696), AUPR (0.9736), recall (0.9005), F1 score (0.9181), and MCC (0.8400) of HiGLDP are the highest compared to those of the other methods. The precision (0.9366) of HiGLDP is slightly lower than gGATLDA with the precision of 0.9381, but the recall (0.8035) of gGATLDA is not balanced with precision, which results in a relatively low F1 score. We can find that deep learning-based models including GANLDA, DMFLDA, GCRFLDA, gGATLDA, VGAELDA, and HiGLDP significantly outperform the inductive matrix completion-based methods SIMCLDA and GMCLDA. This demonstrates that deep neural networks are effective in representation learning. But in deep learning-based models, DMFLDA is a full connected neural network, which is different from the other graph neural network-based methods: HiGLDP, GANLDA, GCRFLDA, gGATLDA, and VGAELDA. From Fig. 3, HiGLDP, GCRFLDA, and gGATLDA performed better than DMFLDA as a whole. But GANLDA and VGAELDA did not perform well in AUPR, F1 score, and MCC. We still can find the superiority of graph neural network in predicting the potential lncRNA-disease associations. Compared with GCRFLDA method, HiGLDP is greatly improved by fusing heterogeneous network information in evaluation indicators.

**Table 2:** Performance of computational models in predicting LncRNA-disease associations

Method	AUC	AUPR	Precision	Recall	F1 score	MCC
HiGLDP	**0.9696**	**0.9736**	0.9366	**0.9005**	**0.9181**	**0.8400**
GANLDA	0.9254	0.5598	0.8427	0.2691	0.4068	0.4667
SIMCLDA	0.6460	0.1620	0.0776	0.5942	0.1372	0.1436
DMFLDA	0.9289	0.9385	0.8819	0.8323	0.8560	0.7206
GCRFLDA	0.9558	0.9567	0.8939	0.8782	0.8859	0.7739
gGATLDA	0.9328	0.9514	**0.9381**	0.8035	0.8652	0.7585
GMCLDA	0.8947	0.2581	0.1532	0.7948	0.2568	0.3049
VGAELDA	0.9539	0.7838	0.8755	0.5719	0.6913	0.6996
GraLTR-LDA	0.7711	0.8327	0.8081	0.6839	0.7404	0.4161
NAGTLDA	0.9121	0.9331	0.8752	0.8377	0.8559	0.7188

Bold values indicate the best performance in each column

**Fig. 4:**
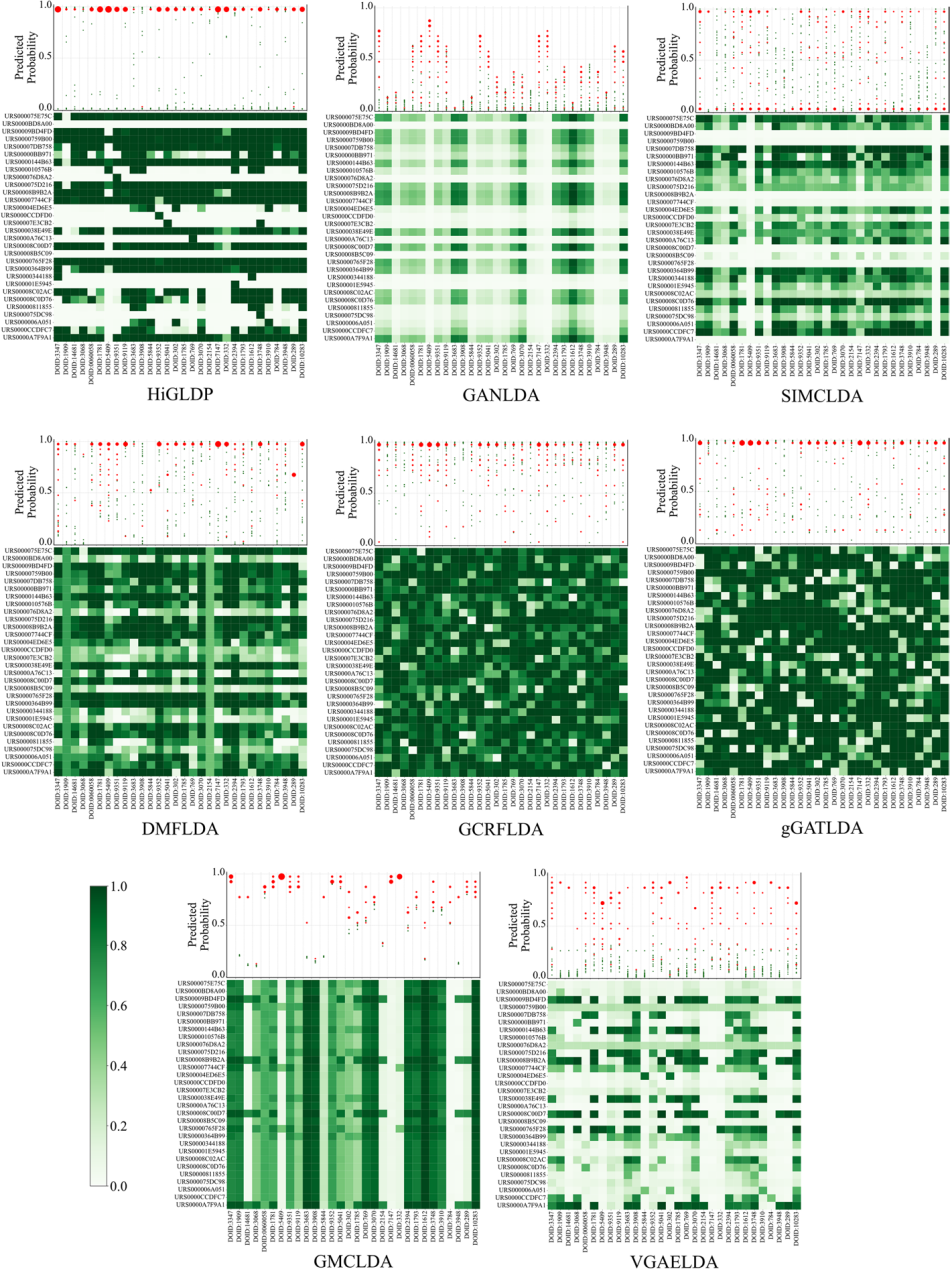
Comparison of predictive probability heatmaps for lncRNA-disease associations across multiple models. It provides a comprehensive visualization of predictive performance across various computational models for predicting lncRNA-disease associations. Each panel features a heatmap illustrating the predicted probabilities of 30 lncRNAs against 30 diseases, where darker shades denote higher probabilities of lncRNA-disease associations. Alongside each heatmap, a scatter plot depicts the predictive performance of each disease across the 30 lncRNAs. Red dots on the scatter plot indicate true-positive labels, representing lncRNAs known to be associated with the disease

Figure 4 visually compares the predictive capabilities of various computational models for lncRNA-disease associations using heatmaps and scatter plots. The heatmaps depict predicted probabilities of associations between 30 lncRNAs and 30 diseases, where darker colors signify higher probabilities. HiGLDP, for instance, shows distinct, dark regions in its heatmap, indicating strong predictive confidence and effective discrimination. In contrast, models like SIMCLDA exhibit less pronounced color variations, suggesting lower ability to differentiate between associated and nonassociated lncRNA-disease pairs. Red dots, the size of which corresponds to the number of overlapping red dots, represent known lncRNA-disease associations. The positioning of these red dots predominantly toward the upper part of the plot, particularly evident in HiGLDP’s scatter plot, indicates accurate predictions with higher probabilities. Conversely, if the red dots are scattered across the middle or lower parts of the plot, as observed in some other models, it suggests less alignment with known lncRNA-disease associations, highlighting potential inconsistencies or inaccuracies in predictions.

### Componential influence analysis through ablation studies

We conducted a series of ablation studies that systematically evaluated different components of its network structure. The leave-one-out validation was performed on each part of the model to assess their respective contributions to the overall performance.

First, we explore the influence of GCN and GAT of our model. Then, we test the effectiveness of the attention mechanism for the representations. In HiGLDP-GCN, we retained the two-layer graph convolutional network along with the attention mechanism but excluded the graph attention network layers. HiGLDP-GAT preserved the graph attention network but omitted the graph convolutional layers. Additionally, we evaluated HiGLDP-NOATT, where the attention mechanism was not utilized, and results relied solely on the outputs from the final network layer. We also conducted additional ablation experiments by evaluating HiGLDP-RFG (only association feature graph) and HiGLDP-CG (only interconnected graph). To explicitly assess whether a simple propagation model could replace the GNN framework, we performed an additional ablation experiment where the GCN–GAT layers were removed, and only RWR-derived features were used (denoted as HiGLDP-RWR).

**Fig. 5:**
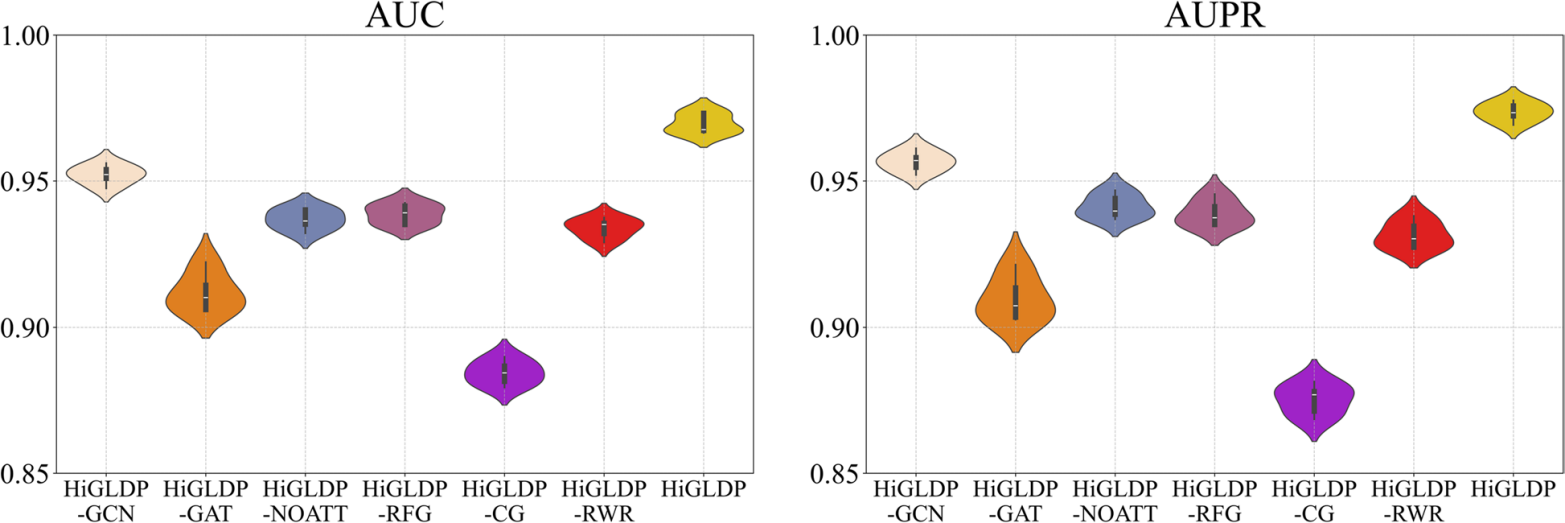
Performance evaluation of HiGLDP and its ablated variants. It provides a comprehensive comparison of its ablated variants using a fivefold cross-validation method. The AUC violin plots illustrate the variability and peak performance of each model. The AUPR violin plots highlight the performance under imbalanced association samples. Individual data values for all performance metrics are provided in Additional file 1

Our findings, as summarized in Table 3, indicate that while HiGLDP-GCN exhibited slightly better performance in AUPR and AUC compared to HiGLDP-GAT and HiGLDP-NOATT, there remains a noticeable gap compared to the complete model. Specifically, HiGLDP recorded an average AUC of 0.9696, AUPR of 0.9736, precision of 0.9366, recall of 0.9005, F1 score of 0.9181, and MCC of 0.8400. As shown in Fig. 5, the AUC violin plot demonstrates that HiGLDP consistently achieves higher median AUC values compared to HiGLDP-GCN, HiGLDP-GAT, and HiGLDP-NOATT. Similarly, the AUPR violin plot shows HiGLDP achieving notably higher scores, highlighting its superior accuracy in identifying true positives and negatives. Either without GCN or GAT modeling, the performance of the model decreases, implying the effectiveness of multimodal graph neural network. After removing the attention mechanism of the channels, it was found that the results also dropped obviously. Furthermore, the attention fusion module in HiGLDP enhances interpretability by quantifying the relative contributions of different information sources. The learned attention weights ${\alpha }_{CG}$ and ${\alpha }_{RFG}$ represent how much each prediction depends on structural connectivity versus feature similarity, allowing us to distinguish whether a lncRNA-disease association is primarily topology-driven or feature-driven. Although our current analysis focuses on predictive performance, these weights can be directly extracted to provide intuitive, view-level explanations for individual predictions.

**Table 3:** Results from variants of our method HiGLDP

Method	AUC	AUPR	Precision	Recall	F1 score	MCC
HiGLDP-GCN	0.9521	0.9566	0.8887	0.8950	0.8918	0.7828
HiGLDP-GAT	0.9118	0.9097	0.8970	0.7392	0.8100	0.6648
HiGLDP-NOATT	0.9369	0.9412	0.8826	0.8753	0.8781	0.7580
HiGLDP-RFG	0.9384	0.9370	0.8756	0.8569	0.8661	0.7352
HiGLDP-CG	0.8841	0.8750	0.8568	0.7505	0.8001	0.6297
HiGLDP-RWR	0.9391	0.9364	0.8711	0.8851	0.8780	0.7540
HiGLDP	**0.9696**	**0.9736**	**0.9366**	**0.9005**	**0.9181**	**0.8400**

Bold values indicate the best performance in each column

**Fig. 6:**
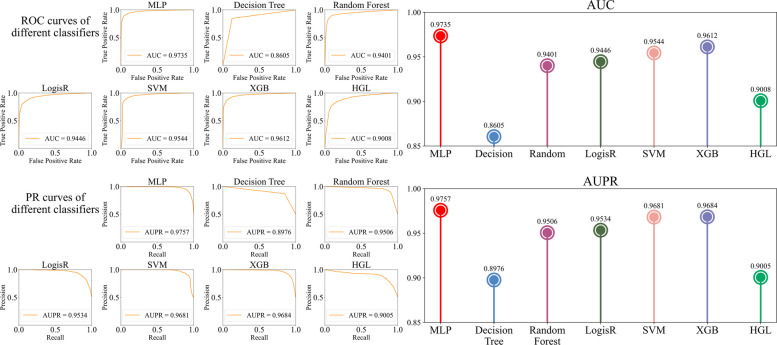
Performance evaluation of different machine learning classifiers used in the HiGLDP model. It provides a comprehensive comparison of different machine learning classifiers using a fivefold cross-validation method. Individual data values for all performance metrics are provided in Additional file 1

We conducted comparative experiments using a range of machine learning classifiers alongside MLP approach. Specifically, MLP achieved an AUC of 0.9735 and an AUPR of 0.9757 (Table 4 and Fig. 6). This performance was closely followed by the XGB, SVM, and LogisR, which also demonstrated high efficacy with AUCs and AUPRs. The recall (0.9097) of LogisR is higher than the MLP with the recall of 0.9095, but the F1 score (0.8896) is lower than the MLP with 0.9218, showing the balance between precision and recall affects the F1 score. Random forest, LogisR, and XGB performed well overall, indicating their strong classification capabilities. As shown in Table 4, the results of random forests are superior to decision trees, reflecting the advantage of random forests in improving accuracy through the ensemble of multiple decision trees. Hypergraph learning (HGL), which performed averagely, is not suitable for this task. These detailed comparisons are visually depicted in Fig. 6, illustrating ROC and PR curves for each classifier. The figures highlight the MLP’s superior performance compared to other classifiers such as SVM, decision tree, and logistic regression. This advantage can be attributed to the high-dimensional, continuous, and nonlinear nature of the node embeddings generated by the hybrid GCN–GAT module. MLP can better model such complex feature interactions through multilayer nonlinear transformations.

**Table 4:** Comparison with different classifiers

Classifier	AUC	AUPR	Precision	Recall	F1 score	MCC
MLP	**0.9735**	**0.9757**	**0.9345**	0.9095	**0.9218**	**0.8465**
Decision tree	0.8605	0.8976	0.8727	0.8358	0.8538	0.7143
Random forest	0.9401	0.9506	0.9224	0.8852	0.9034	0.8113
LogisR	0.9446	0.9534	0.8714	**0.9097**	0.8896	0.7757
SVM	0.9544	0.9681	0.9266	0.8894	0.9072	0.8193
XGB	0.9612	0.9684	0.9182	0.8867	0.9020	0.8080
HGL	0.9008	0.9005	0.8627	0.8051	0.8328	0.6782

Bold values indicate the best performance in each column

### Sensitivity analysis of multi-omic integration

To further examine the contribution of each omic layer and evaluate the sensitivity of HiGLDP to multi-omic integration, we performed an ablation analysis in which one omic dataset was removed at a time while keeping all other settings identical. The full model integrates seven omic sources, including four lncRNA-related (lncRNA–mRNA, lncRNA–RBP, lncRNA–protein, and lncRNA–disease) and three disease-related (disease–metabolite, disease–circRNA, and disease ontology) networks. Each variant was denoted according to the omitted dataset.

As summarized in Table 5, removing any omic layer led to a noticeable reduction in predictive performance, confirming that each omic source provides unique and complementary biological information. The most significant performance decline occurred when excluding the disease ontology (DO) layer (AUC = 0.8888, AUPR = 0.8939), highlighting its essential role in capturing semantic and functional relationships among diseases. By contrast, the exclusion of individual lncRNA-related omic layers caused smaller degradations, likely due to the redundancy among multiple lncRNA-centered networks.

**Table 5:** Sensitivity analysis of HiGLDP performance to the exclusion of individual omic layers

Data source	AUC	AUPR	Precision	Recall	F1 score	MCC
Without LM	0.9362	0.9277	0.8771	0.8598	0.8683	0.7655
Without LR	0.9419	0.9405	0.9022	0.8399	0.8698	0.7504
Without LP	0.9355	0.9307	0.9092	0.8283	0.8666	0.7484
Without LD	0.9305	0.9306	0.8389	0.8786	0.8557	0.7360
Without DM	0.9200	0.9225	0.8907	0.8240	0.8558	0.7785
Without DC	0.9182	0.9232	0.9033	0.8205	0.8598	0.7355
Without DO	0.8888	0.8939	0.7624	0.8737	0.8133	0.6054
Full data	**0.9696**	**0.9736**	**0.9366**	**0.9005**	**0.9181**	**0.8400**

Bold values indicate the best performance in each column

A comparison between the two omic categories revealed that removing disease-related layers generally produced greater degradation than removing lncRNA-related ones. This suggests that a balanced representation between lncRNA and disease features is critical for achieving stable and robust inference. Overall, these results confirm that HiGLDP’s predictive strength arises from the synergistic integration of heterogeneous omic information, which allows the model to capture multi-pathway and multi-mechanism interactions underlying lncRNA-disease associations.

### Model validation through disease-specific case analyses

We selected osteosarcoma (OS), melanoma, and glioblastoma multiforme (GBM) as representative case studies to evaluate the generalizability of HiGLDP across distinct biological contexts. These three cancers were chosen not based on model optimization but because they represent diverse tissue origins (bone, skin, and central nervous system) and have relatively well-documented lncRNA annotations in public databases, facilitating validation of predicted associations. We used our model to predict potential lncRNA-disease associations where these associations were previously unknown, ranking the lncRNAs based on the predicted scores. We selected the top 20 lncRNAs for each disease as predicted by HiGLDP and searched for experimental evidence in RNAcentral, LncRNADisease v3.0 database [27], and RNADisease V4.0 [28].

Osteosarcoma (OS) is the most common histological form of primary bone cancer, most prevalent in children and adolescents [29]. As shown in Table 6, all the top 20 predicted associated lncRNAs have been confirmed by the databases and literature. For instance, the deletion of lncRNA URS0000812019 (SPRY4-IT1), ranked fourth, was shown to significantly inhibit OS cell proliferation by causing G1 arrest and promoting apoptosis [30].

**Table 6:** Top ranked diseases-related lncRNAs predicted by HiGLDP

Disease	Rank	LncRNA	Evidence
**Osteosarcoma**	1	URS0000774BAA	PMID:
	2	URS000075DAEC	PMID: 29,416,922
	3	URS000076B6DF	PMID: 29,416,922
	4	URS0000812019	PMID: 28,078,006;31,746,422
	5	URS0000776303	PMID: 29,416,922
	6	URS0000204428	PMID: 29,416,922
	7	URS000076BEC3	PMID: 29,416,922
	8	URS00001328A6	PMID: 28,409,547;29,384,226
	9	URS000050F1EF	PMID: 28,409,547;29,384,226
	10	URS0000A8B321	PMID: 29,246,789;31,898,343
	11	URS00000E70EF	PMID: 28,409,547;29,384,226
	12	URS0000A887DA	PMID: 28,409,547;29,384,226
	13	URS00005D94B6	PMID: 29,246,789; 31,898,343
	14	URS00005303E4	PMID: 28,409,547;29,384,226
	15	URS000075B620	PMID: 29,194,827;29,257,211
	16	URS00003777D5	PMID: 29,246,789;31,898,343
	17	URS000075D95B	PMID: 28,409,547;29,384,226
	18	URS000075ADFF	PMID: 28,549,102
	19	URS00002F26AC	PMID: 29,246,789;31,898,343
	20	URS00000BB971	PMID: 28,409,547;29,384,226
**Melanoma**	1	URS0000774BAA	PMID: 31,173,352;33,202,380;31,462,890
	2	URS000075DAEC	PMID: 31,173,352;31,462,890;32,096,166
	3	URS000076B6DF	PMID: 31,173,352;31,462,890;32,096,166
	4	URS0000776303	PMID: 31,173,352;31,462,890;32,096,166
	5	URS0000204428	PMID: 31,173,352;31,462,890;32,096,166
	6	URS000076BEC3	PMID: 31,173,352;31,462,890;32,096,166
	7	URS00002C6CC0	PMID: 27,016,304
	8	URS0000364B99	PMID: 27,016,304
	9	URS000038E49E	PMID: 27,016,304
	10	URS00000EF56E	PMID: 27,016,304
	11	URS00001328A6	PMID: 27,016,304
	12	URS00005BCD38	PMID: 25,700,553
	13	URS00005FEBEE	PMID: 27,016,304
	14	URS000075DE90	-
	15	URS000075ADFF	PMID: 28,409,554
	16	URS00003777D5	PMID: 30,257,602
	17	URS000075F11A	-
	18	URS00007681C7	PMID: 26,169,368;27,043,545
	19	URS000075F0E1	-
	20	URS000075AF07	PMID: 27,016,304
**Glioblastoma multiforme**	1	URS0000774BAA	PMID: 33,057,597
	2	URS000075DAEC	PMID: 33,057,597
	3	URS000076B6DF	PMID: 33,057,597
	4	URS0000776303	PMID: 33,057,597
	5	URS0000364B99	PMID: 22,234,798;25,378,224;26,111,795
	6	URS000004D655	PMID: 23,046,790;27,306,825
	7	URS00000EF56E	PMID: 22,234,798;25,378,224;26,111,795
	8	URS000075B87C	PMID: 23,046,790;27,306,825
	9	URS0000CCE0B6	PMID: 23,726,844
	10	URS0000204428	PMID: 33,057,597
	11	URS000075DF55	PMID: 23,046,790;27,306,825
	12	URS00005FEBEE	PMID: 22,234,798;25,378,224;26,111,795
	13	URS000076BEC3	PMID: 33,057,597
	14	URS000075B0B8	PMID: 23,046,790;27,306,825
	15	URS000075DDC7	PMID: 23,046,790;27,306,825
	16	URS00008B991F	PMID: 23,726,844
	17	URS000075A540	PMID: 23,046,790;27,306,825
	18	URS000038E49E	PMID: 22,234,798;25,378,224;26,111,795
	19	URS0000732D5D	PMID: 23,046,790;27,306,825
	20	URS000075A92F	PMID: 23,046,790;27,306,825

“-” means no relevant evidence was found

Melanoma is the most aggressive and deadliest form of skin cancer [31]. It indicates that 17 lncRNAs predicted to be associated with melanoma have been validated. The second-ranked lncRNA URS000075DAEC (NEAT1) was confirmed to be associated with melanoma in recent databases. Functional assays demonstrated that overexpression of NEAT1 promoted the proliferation, migration, and invasion of melanoma cells, while NEAT1 knockdown significantly restrained melanoma cell progression [32].

Glioblastoma multiforme (GBM) is the most common primary intracranial tumor, accounting for 40–50% of brain tumors [33]. An increasing number of lncRNAs have been reported to be associated with glioblastoma multiforme. In a study by Bi et al. [34], quantitative real-time PCR (qRT-PCR) showed that NEAT1 (URS000075DAEC) was upregulated in serum samples of glioblastoma multiforme patients and glioma stem cells (GSCs) isolated from U87 and U251 cell lines. Functional experiments showed that NEAT1 knockdown restrained malignant behaviors of GSCs, including proliferation, migration, and invasion. Table 6 lists the top 20 predicted lncRNAs associated with glioblastoma multiforme, all of which have been validated to be associated with the disease. These case studies validate the efficacy of HiGLDP in identifying novel lncRNA-disease associations.

## Discussion

The results of this study demonstrate that HiGLDP effectively integrates heterogeneous biological information and advanced graph-based architectures. Compared with state-of-the-art computational methods, HiGLDP consistently achieved superior performance across multiple evaluation metrics, highlighting the advantages of combining stratified interaction graphs, hybrid GCN–GAT layers, and attention-based feature fusion. The ablation experiments further confirmed that each component of the framework contributed meaningfully to the final performance, underscoring the importance of simultaneously modeling local and global topological structures in biological networks.

From a biological perspective, the case studies on osteosarcoma, melanoma, and glioblastoma multiforme illustrate the practical utility of HiGLDP in uncovering novel and biologically relevant lncRNA-disease associations. Many of the top-ranked predictions have already been validated in independent experimental studies, providing strong evidence for the model’s predictive reliability. Such findings emphasize that HiGLDP can not only recapitulate known biology but also serve as a discovery tool to guide further functional validation in experimental settings. Moreover, although many of the top-ranked associations predicted by HiGLDP were subsequently validated in curated databases, this consistency reflects the model’s reliability rather than a mere reproduction of prior knowledge. The strict cross-validation strategy ensured that all verified associations were excluded from the training process, demonstrating that HiGLDP captures transferable biological patterns rather than memorizing existing data. Furthermore, several high-confidence predictions without current literature support may represent previously unrecognized lncRNA-disease associations, providing promising hypotheses for future experimental verification.

Despite these strengths, several limitations should be acknowledged. First, although multi-omic data were incorporated, the model still depends on the availability and quality of curated association databases, which remain incomplete and biased toward well-studied lncRNAs and diseases. Second, while the attention mechanism enhances interpretability, the model does not yet provide fully transparent biological explanations for the inferred associations. Third, the current framework was primarily evaluated on benchmark datasets; its ability to generalize to entirely novel lncRNAs or rare diseases requires further validation.

Future work may focus on integrating additional omics layers to capture more comprehensive regulatory contexts. HiGLDP inherently supports finer-grained interpretability within each graph. The GAT layers compute neighbor-level attention coefficients that quantify how much each neighboring node contributes to the message-passing process. While these coefficients are not visualized in the current work, they could be analyzed in future studies to identify the most influential lncRNAs or diseases for each prediction, offering case-specific insights into the underlying biological relationships. Moreover, coupling HiGLDP with causal inference techniques or explainable AI approaches could enhance biological interpretability, facilitating its application in translational research. Expanding the model to incorporate temporal or spatial information, such as dynamic expression patterns from disease progression or tissue-specific contexts [35], could further improve prediction accuracy and biological relevance. Finally, prospective collaboration with experimental studies will be critical to validate novel predictions and explore their potential as diagnostic biomarkers or therapeutic targets.

## Conclusions

In this study, we introduce HiGLDP, a novel computational framework designed to enhance the prediction of lncRNA-disease associations by integrating multi-omic data with advanced graph-based deep learning techniques. By leveraging genomic, transcriptomic, and proteomic data, HiGLDP constructs comprehensive similarity networks for lncRNAs and diseases, which are further refined through RWR and DAE. The transformation of the bipartite graph into an interconnected graph, combined with the construction of an association feature graph using cosine similarity, allows HiGLDP to capture intricate lncRNA-disease interactions effectively. Comprehensive evaluations, including fivefold and tenfold cross-validation, demonstrate that HiGLDP significantly outperforms existing models in terms of AUC, AUPR, precision, recall, F1 score, and MCC. Case studies further validate the model’s ability to predict novel lncRNA-disease associations, underscoring its potential for real-world applications. Future work could focus on improving the model’s ability to generalize to datasets with unknown associations and integrating additional biological information to enhance predictive accuracy. In conclusion, by integrating multi-omic data and employing advanced graph neural network techniques, HiGLDP offers a robust, scalable, and interpretable framework that advances our understanding of lncRNA-disease interactions.

## Key points

- HiGLDP introduces a stratified interaction graph framework that reformulates lncRNA–disease prediction from node-level modeling to association-level representation, enabling the capture of high-order and cross-association dependencies that conventional bipartite graphs cannot express.
- A hybrid GCN–GAT architecture with attention-guided feature fusion adaptively balances structural and semantic information, enhancing both predictive accuracy and interpretability.
- The model systematically integrates genomic, transcriptomic, and proteomic data through random walk with restart and denoising autoencoder modules, achieving robust multi-omic representations and reducing data sparsity effects.
- Extensive evaluations demonstrate that HiGLDP delivers substantial methodological and performance advances over existing GNN-based frameworks, establishing it as a unified and interpretable model for lncRNA-disease association prediction.

## Methods

### Datasets

In our study, we integrated multiple databases to support our research on the associations between lncRNAs and diseases. RNAcentral [36] is a database that compiles ncRNA sequences from various specialized resources, assigning unique identifiers to each distinct RNA sequence. As different lncRNA databases lack a unified identity number, we used identifiers from RNAcentral as unified labels for lncRNAs to facilitate the smooth progression of our research.

The Disease Ontology (DO) database [37] focuses on representing both common and rare disease concepts, aiming to provide an open-source ontology for integrating biomedical data related to human diseases. Each node in DO represents one disease term, organized in a directed acyclic graph (DAG) with “IS_A” relationships. MEDIC, part of the Comparative Toxicogenomics Database (CTD) [38], integrates terms, synonyms, and identifiers from Online Mendelian Inheritance in Man (OMIM) [39] with terms, synonyms, definitions, identifiers, and hierarchical relationships from the Medical Subject Headings (MeSH) [40]. In this study, we mapped diseases related to lncRNAs using terms and synonyms from both DO and MEDIC.

LncRNADisease [41] is a database that curates experimentally supported lncRNA-disease association data. In this study, we used the association data provided by this database as benchmark data. starBase v2.0 [42] provides datasets related to lncRNA, including associations between lncRNAs and RNA-binding proteins (RBP), mRNAs, and proteins, which are used to establish comprehensive lncRNA association networks. Furthermore, to assess the similarity of disease terms from the perspective of metabolites, we adopted an annotation method from the literature to establish links between diseases and metabolites through the Human Metabolome Database (HMDB) [43]. Similarly, we assessed the relationship between diseases and circRNAs using circRNADisease [44], Circ2Disease [45], and circR2Disease [46], which include known human circRNA-disease associations.

To ensure a fair and unbiased comparison across all baseline methods, we used exactly the same dataset and data partitioning for model training and evaluation. All methods were trained on identical sets of positive and negative lncRNA-disease pairs with a 1:1 ratio and evaluated using both fivefold and tenfold cross-validation, where the test associations were strictly excluded from the training folds. The source code and processed datasets supporting this study are publicly available via Zenodo (10.5281/zenodo.18522355) [47].

### Multi-omic feature encoding for lncRNAs and diseases

#### LncRNA similarity network

There are numerous types of data related to lncRNAs, and we have thoroughly considered the similarity of lncRNAs from multiple perspectives. Firstly, we examined the relationship between lncRNAs and diseases, using the Gaussian kernel function to calculate the similarity between lncRNAs.

The lncRNA-disease association is represented as A, where ${A}_{ij}=1$ if there is a known association between lncRNA ${l}_{i}$ and disease ${d}_{j}$. Therefore, we utilized the Gaussian interaction profile kernels to calculate the similarity $GIP({l}_{i}, {l}_{j})$ between lncRNA ${l}_{i}$ and lncRNA ${l}_{j}$. The calculation is as follows:

2 $$\mathrm{GIP}\left({\mathrm{l}}_{\mathrm{i}}, {\mathrm{l}}_{\mathrm{j}}\right)=\mathrm{exp}\left(-\frac{{\Vert {\mathrm{A}}_{\mathrm{i}}-{\mathrm{A}}_{\mathrm{j}}\Vert }^{2}}{{\upsigma }^{2}}\right)$$

where ${A}_{i}$ and ${A}_{j}$ are the interaction profiles of lncRNA ${l}_{i}$ and ${l}_{j}$, respectively, and $\sigma$ is a parameter that controls the width of the Gaussian function.

In addition to diseases, lncRNAs are associated with various omics data. These association data can be considered bipartite graphs, recording the relationships between lncRNAs and other biological entities, where an association is denoted as 1 and no association as 0. For instance, in the lncRNA-mRNA data, if lncRNA ${l}_{i}$ is associated with mRNA ${m}_{j}$, then ${M}_{ij}=1$; otherwise, ${M}_{ij}=0$. The shared mRNA set was obtained by calculating the intersection of the lncRNA-mRNA sets, and the Jaccard similarity was employed to measure the similarity of the mRNA sets. A larger shared mRNA set indicates that lncRNAs share more mRNAs, signifying higher similarity. The shared mRNA similarity between two lncRNAs is represented by Jaccard similarity coefficient.

#### Disease similarity network

To evaluate the similarities among various diseases associated with lncRNAs, we employ the FNSemSim approach [48], a method we previously developed. FNSemSim integrates functional and semantic similarities of diseases to calculate comprehensive similarity scores. After computing pairwise disease similarities, we apply min–max normalization to appropriately scale these values.

For any two diseases, ${d}_{1}$ and ${d}_{2}$, within the set *D* of disease terms related to lncRNAs, the similarity $FNSemSim({d}_{1}, {d}_{2})$ is computed using our method. Using these similarity scores, we construct a disease similarity network (DFS), which specifically highlights the connections between diseases associated with lncRNAs.

Additionally, we extend our analysis to consider the relationships between disease ontology (DO) terms and circRNAs, as well as between DO terms and metabolites. To further enrich the disease similarity network, we calculate disease similarity at the levels of disease-related metabolites and circRNAs using the Jaccard similarity coefficient.

#### Disease and LncRNA feature learning

Upon obtaining the similarity networks from the heterogeneous network, node embeddings within each similarity network are generated using RWR method [49]. The RWR algorithm is formulated as follows:

3 $$\mathrm{r}=\mathrm{cWr}+(1-\mathrm{c})\mathrm{e}$$

where *c* is a constant within the range (0, 1), *W* represents the transition probability matrix, *e* is the starting vector with *e*[*i*] = 1 if *i* is the starting node and *e*[*i*] = 0 otherwise, and *r* is a column vector. Leveraging the topological relationships between each disease or lncRNA and other nodes in the network, probability distributions are derived through multiple iterations and convergence of RWR. By aggregating the probability distributions of all nodes, a representative information for a specific node is formed. Ultimately, feature representations for diseases and lncRNAs are obtained. We used DAE to further extract the features of lncRNA and disease, respectively.

### Stratified interaction graphs for lncRNA-disease associations

#### Interlacing lncRNA-disease dyads into interconnected graphs

We convert the bipartite graph of lncRNA-disease associations into an interconnected graph containing lncRNA-disease relationship nodes by defining these associations as nodes. The association node between lncRNA $L$ and disease $D$ can be defined as ${PN}_{L,D}$.

To construct the interconnected graph, we apply the principle that if two nodes share a common lncRNA or a common disease, there is an edge between them. Therefore, for nodes ${PN}_{L,D}$ and ${PN}_{{L}^{*},{D}^{*}}$, their connectivity can be defined as follows:

4 $$\mathrm{CG}\left({\mathrm{PN}}_{\mathrm{L,D}},{\mathrm{PN}}_{{\mathrm{L}}^{*},{\mathrm{D}}^{*}}\right)=\left\{\begin{array}{c}1, \mathrm{L}={\mathrm{L}}^{*} or \mathrm{D}={\mathrm{D}}^{*}\\ 0, \mathrm{else}\end{array}\right.$$

By leveraging the connectivity of relationship nodes between lncRNA $L$ and disease $D$, we create an interconnected graph that encompasses their associations.

#### Composite feature graph assembly for lncRNA and disease

Furthermore, we concatenate the features of lncRNAs and diseases as the features of the association nodes. The cosine similarity is applied to construct the association feature graph of lncRNAs and diseases. For any lncRNA-disease relationship node, we calculate the cosine similarity between its feature vector and those of other nodes. The similarity between nodes ${PN}_{i}$ and ${PN}_{j}$ is defined as follows:

5 $$\mathrm{RFG}\left({\mathrm{PN}}_{\mathrm{i}},{\mathrm{PN}}_{\mathrm{j}}\right)=\frac{F\left({\mathrm{PN}}_{\mathrm{i}}\right)\cdot \mathrm{F}\left({\mathrm{PN}}_{\mathrm{j}}\right)}{\Vert \mathrm{F}\left({\mathrm{PN}}_{\mathrm{i}}\right)\Vert \cdot \Vert \mathrm{F}\left({\mathrm{PN}}_{\mathrm{j}}\right)\Vert }$$

where $F\left({PN}_{i}\right)$ and $F\left({PN}_{i}\right)$ represent the association features of nodes ${PN}_{i}$ and ${PN}_{j}$, respectively. For each lncRNA-disease association node, we identify the most similar node as its neighbors, thereby constructing the association feature graph of lncRNA and disease.

### Dimensional feature refinement via graph-based neural architectures

#### Unraveling hierarchical structures with convolutional graph learners

The feature extraction process involves a three-layer hybrid graph neural network architecture, which consists of two graph convolutional layers and a graph attention layer. The graph convolutional network (GCN) is designed to learn node representations by leveraging the topological structure of the graph. The GCN layer can be described by the following equation:

6 $${\mathrm{H}}_{\mathrm{GCN}}^{\left(\mathrm{l}+1\right)}=\sigma ({\widetilde{\mathrm{D}}}^{-\frac{1}{2}}\widetilde{\mathrm{A}}{\widetilde{\mathrm{D}}}^{-\frac{1}{2}}{\mathrm{H}}^{(\mathrm{l})}{\mathrm{W}}^{(\mathrm{l})})$$

where ${H}^{(l)}$ is the matrix of node features at layer *l*, $\widetilde{A}$ is the adjacency matrix of the graph with added self-connections, $\widetilde{D}$ is the degree matrix of $\widetilde{A}$, ${W}^{(l)}$ is the weight matrix for layer* l*, and $\sigma$ is a nonlinear activation function.

The GAT layer computes node features by incorporating the mechanism of attention, which allows the model to focus on more important nodes within a neighborhood. The output of the GAT layer is given by the following:

7 $${\mathrm{h}}_{\mathrm{i}}^{(\mathrm{l}+1)}=\upsigma \left({\sum }_{\mathrm{j}\in \mathcal{\mathrm{N}}(\mathrm{i})}{\upalpha }_{\mathrm{ij}}^{(\mathrm{l})}{\mathrm{W}}^{(\mathrm{l})}{\mathrm{h}}_{\mathrm{j}}^{(\mathrm{l})}\right)$$

where ${h}_{i}^{(l)}$ and ${h}_{j}^{(l)}$ are the feature vectors of nodes *i* and *j* at layer *l*, ${\alpha }_{ij}^{(l)}$ is the attention coefficient between nodes *i* and *j*, which determines the importance of node *j*’s features to node *i*, and ${W}^{(l)}$ is the weight matrix at layer *l*.

To effectively combine the advantages of GCN and GAT, we adopt GCN-GAT-GCN for feature extraction as follows:

8 $$\left\{\begin{array}{c}{\mathrm{H}}_{\mathrm{GCN}}^{(1)}=\mathrm{GCN}(\mathrm{A},{\mathrm{H}}_{\mathrm{c}}^{\left(0\right)})\\ {\mathrm{H}}_{\mathrm{GAT}}^{(2)}=\mathrm{GAT}(\mathrm{A},{\mathrm{H}}_{\mathrm{GCN}}^{\left(1\right)})\\ {\mathrm{H}}_{\mathrm{GCN}}^{(3)}=\mathrm{GCN}(\mathrm{A},{\mathrm{H}}_{\mathrm{GAT}}^{\left(2\right)})\end{array}\right.$$

where $A$ represents the adjacent matrix of the interconnected graph $CG$ or the association feature graph $RFG$ and ${H}^{l}$ represents the output of the layer $l$. Finally, we concatenate the ${H}_{GCN}^{(1)}$ and ${H}_{GCN}^{(3)}$ to get the representations of $PN$ nodes in the two graphs $CG$ and $RFG$, respectively, as follows:

9 $$\mathrm{F}={\mathrm{H}}_{\mathrm{GCN}}^{\left(1\right)}\oplus {\mathrm{H}}_{\mathrm{GCN}}^{\left(3\right)}$$

The resulting feature representations, which are rich in both local and global graph structure information, are integrated by GCN layer 1 and layer 3. This integration makes them highly effective for downstream tasks. The final *K*-dimensional features of the relationship node $PN$ can be extracted in these hybrid graph neural networks and denoted as ${F}_{CG}\in {R}_{CG}^{{N}_{PN}*K}$ and ${F}_{RFG}\in {R}_{RFG}^{{N}_{PN}*K}$.

The described hybrid approach ensures that the model not only captures the structural and feature-based properties of the nodes but also adapts to the specific importance of neighboring nodes.

#### Illuminating functional networks with attention-focused graph learners

To further enhance the interpretability of the model, we used an attention mechanism to fuse all the representations of the interconnected graph $CG$ or the association feature graph $RFG$. The two representations of node ${PN}_{i}$ can be denoted by ${H}_{CG}^{i}$ and ${H}_{RFG}^{i}$. Let vector ${\alpha }^{i}$ represent their attention vector, which can be obtained by the attention function $Attention({H}_{CG}^{i}, {H}_{RFG}^{i})$:

10 $${\upalpha }^{\mathrm{i}}\left({\upalpha }_{\mathrm{CG}}^{\mathrm{i}},{\upalpha }_{\mathrm{RFG}}^{\mathrm{i}}\right)=\mathrm{Attention}({\mathrm{H}}_{\mathrm{CG}}^{\mathrm{i}},{\mathrm{H}}_{\mathrm{RFG}}^{\mathrm{i}})$$

where ${\alpha }_{CG}^{i}$ and ${\alpha }_{RFG}^{i}$ are the corresponding attention scores of ${H}_{CG}^{i}$ and ${H}_{RFG}^{i}$. For the definition of attention function, we first compute a weight score ${w}_{CG}^{i}$ for ${H}_{RFG}^{i}$ as follows:

11 $${\mathrm{w}}_{\mathrm{CG}}^{\mathrm{i}}={\mathrm{q}}^{\mathrm{T}}\bullet \mathrm{tanh}(\mathrm{W}\bullet {\left({\mathrm{H}}_{\mathrm{CG}}^{\mathrm{i}}\right)}^{\mathrm{T}}+\mathrm{b})$$

where $W\in {\mathbb{R}}^{{d}_{l}\times {d}_{h}}$ is a weight matrix, $b\in {\mathbb{R}}^{{d}_{l}\times 1}$ is a bias vector, and $q\in {\mathbb{R}}^{{d}_{l}\times 1}$ is a shared attention vector. ${d}_{l}$ is the dimension of attention layer. ${d}_{h}$ is the dimension of final representation of ${PN}_{i}$ learned from convolutional graph learners. Through the same operation, we can also obtain the values ${w}_{RFG}^{i}$ of node $i$ for ${H}_{RFG}^{i}$.

Then, the obtained weight values are normalized by $softmax$ function as attention scores. Consequently, we can use the following equation to compute ${\alpha }_{CG}^{i}$:

12 $${\upalpha }_{\mathrm{CG}}^{\mathrm{i}}=\mathrm{softmax}\left({\mathrm{w}}_{\mathrm{CG}}^{\mathrm{i}}\right)=\frac{\mathrm{exp}({\mathrm{w}}_{\mathrm{CG}}^{\mathrm{i}})}{\mathrm{exp}\left({\mathrm{w}}_{\mathrm{CG}}^{\mathrm{i}}\right)+\mathrm{exp}({\mathrm{w}}_{\mathrm{RFG}}^{i})}$$

Similarly, ${\alpha }_{RFG}^{i}$ can be calculated. Therefore, the final representation of ${PN}_{i}$ is defined by weighted summation of ${H}_{CG}^{i}$ and ${H}_{RFG}^{i}$.

13 $${\mathrm{H}}^{\mathrm{i}}={\upalpha }_{\mathrm{CG}}^{\mathrm{i}}\bullet {\mathrm{H}}_{\mathrm{CG}}^{\mathrm{i}}+{\upalpha }_{\mathrm{RFG}}^{\mathrm{i}}\bullet {\mathrm{H}}_{\mathrm{RFG}}^{\mathrm{i}}$$

Finally, we employ a multilayer perceptron (MLP) as the classification model, which consists of an input layer, hidden layers, and an output layer. The input to the MLP is the ${PN}_{i}$ representation vector ${H}^{i}$, and the output is the probability that the lncRNA-disease pair is related.

## Supplementary Information

Additional file 1: Table S1. The fivefold Cross-Validation Testing Results of Computational Models in Predicting LncRNA-disease associations. Table S2. The fivefold Cross-Validation Testing Results from variants of HiGLDP. Table S3. The fivefold Cross-Validation Testing Results of Different Classifiers. Table S4. The fivefold Cross-Validation Testing Results of HiGLDP Performance to the Exclusion of Individual Omic Layers.

## Data Availability

All data generated or analysed during this study are included in this published article, its supplementary information files and publicly available repositories. The source code and processed datasets are available at Zenodo ([https://doi.org/10.5281/zenodo.18522355] (https://doi.org/10.5281/zenodo.18522355)), with the development repository maintained at GitHub ([https://github.com/St0neWang/HiGLDP] (https://github.com/St0neWang/HiGLDP)). Individual data values for all performance metrics are provided in Additional file 1.

## Abbreviations

LncRNA: Long noncoding RNA
RWR: Random walk with restart
DAE: Denoising autoencoders
GCN: Graph convolutional network
GAT: Graph attention network
MLP: Multilayer perceptron
GIP: Gaussian interaction profile
MCC: Matthews correlation coefficient
FPR: False-positive rate
TPR: True-positive rate
PR: Precision recall
AUPR: Area under the PR curve
ROC: Receiver operating characteristic curve
AUC: Area under the ROC curve
OS: Osteosarcoma
GBM: Glioblastoma multiforme
GSC: Glioma stem cell
